# Association of IL-10 Gene Polymorphism With IL-10 Secretion by CD4 and T Regulatory Cells in Human Leprosy

**DOI:** 10.3389/fimmu.2020.01974

**Published:** 2020-08-06

**Authors:** Mohammad Tarique, Huma Naz, Chaman Saini, Mohd Suhail, Hari Shankar, Neena Khanna, Alpana Sharma

**Affiliations:** ^1^Department of Biochemistry, All India Institute of Medical Sciences (AIIMS), New Delhi, India; ^2^Centre for Interdisciplinary Research in Basic Sciences, Jamia Millia Islamia, New Delhi, India; ^3^King Fahd Medical Research Center, King Abdulaziz University, Jeddah, Saudi Arabia; ^4^Department of Medical Laboratory Technology, Faculty of Applied Medical Sciences, King Abdulaziz University, Jeddah, Saudi Arabia; ^5^Parasite-Host Biology Group, ICMR – National Institute of Malaria Research, New Delhi, India; ^6^Department of Dermatovenerology, All India Institute of Medical Sciences (AIIMS), New Delhi, India

**Keywords:** cytokines, gene polymorphisms, leprosy, interleukin-10, regulatory T-cells, helper T-cells

## Abstract

Leprosy is a chronic bacterial disease caused by *Mycobacterium leprae*. Cytokines are known to play vital role as a peacekeeper during inflammatory and other immunocompromised conditions such as leprosy. This study has tried to bridge the gap of information on cytokine gene polymorphisms and its potential role in the pathogenesis of leprosy. Interleukin-10 (IL-10) is an immunosuppressive cytokine, found to be elevated in leprosy that accounted for the suppression of host’s immune system by regulating the functions of other immune cells. T helper cells and T regulatory (Tregs) cells are the major source of IL-10 in lepromatous leprosy patients. In this study, we have documented the association of IL-10 cytokine gene polymorphism with the disease progression. A total of 132 lepromatous leprosy patients and 120 healthy controls were analyzed for IL-10 cytokine gene polymorphisms using PCR-SSP assay and flow cytometry was used to analyze IL-10 secretion by CD4 and Tregs in various genotype of leprosy patients. The frequencies of IL-10 (-819) TT and IL-10 (-1082) GG genotypes were significantly higher in leprosy patients as compared to healthy controls. This observation advocates that these genotypes were associated with the susceptibility and development of the disease. In addition, flow cytometry analysis demonstrated an increased number of IL-10 producing CD4 and Treg cells in IL-10 (819) TT genotype compared to CT and CC genotypes. These observations were further supported by immunohistochemical studies. Therefore, we can conclude that IL-10 cytokine gene polymorphisms by affecting its production can determine the predilection and progression of leprosy in the study population.

## Introduction

Leprosy is a bacterial disease caused by *Mycobacterium leprae* (*M. leprae*), which mainly affects macrophages and Schwann cells from the peripheral nerves. It offers an ideal model to study the host pathogen interaction and immune dysregulation in human because each clinical form of the disease is accompanied with the diverse levels of immunological alterations during *M. leprae* infection (1). Leprosy disease is mainly classified into two forms, paucibacillary (PB) and multibacillary (MB). PB pole is associated with a strong cell-mediated immunity (CMI), and it is relatively resistant to the pathogens and localized infections. While, MB pole is characterized by the defective cell-mediated immune response comprising foamy macrophages in the dermis due to very high number of bacilli accompanied with the lesions all over the body parts. In between two poles of the disease, borderline forms of tuberculoid and lepromatous disease are laid which are immunologically unstable (2–4).

Cytokines play a vital role in activating host-pathogen interaction and in the immunopathogenesis of leprosy (2). PB form of the disease commencement correlates with Th1 (IL-2, IL-12, TNF-α, IFN-γ) cytokines with a localized infection but vigorous cell mediated immune response; whereas, MB form is associated with a Th2 and Th3 cytokine (IL-10, TGF-β, IL-4) response, presence of local skin lesions, prominent antibody production, but very weak CMI response (5). IL-10 and TGF-β appeared as the sole factor puts forth an anti-proliferative effect on Th1 and Th2 cells by perturbing the differentiation of either Th1 or Th2 cells and inhibition of pro-inflammatory cytokine production, that eventually result into the progression of Th3 immune response (6, 7). Our laboratory have previously reported the association of increased levels of IL-10 in lepromatous leprosy patients with high bacterial load of *M. leprae* and suppressed immune system of the host (2, 6). Furthermore, an increased frequency of IL-35 producing Tregs was observed in lepromatous leprosy patients, which is associated with the disease severity (8). Treg cells plays vital role in leprosy pathogenesis and fate of these cells also depends on cytokine milieu. This is supported from our previous study that explored the changes in the plasticity of Tregs under the influence of IL-12 and IL-23 treatment (9). Besides Tregs, another immunosuppressive population of γδ T cells plays an important role in the pathogenesis of leprosy (10, 11). IL-10, secreted by monocytes/macrophage, T cell subsets (Th2, Tregs) and B regulatory cells shows immune regulatory effects in case of human leprosy (12). IL-10-producing regulatory B cells transformed T effector cells into Tregs, thus enhanced regulatory T cells functions in human leprosy. Also, high levels of IL-10 are reported in lepromatous polar form compared with tuberculoid polar form and a low TNF-α/IL-10 ratio was found associated with disease progression (13). Single nucleotide polymorphisms (SNPs) are thought to be one of the most abundant causes of genomic variation in humans. The presence of SNPs in a gene can create differences in the structure and functions of a coded protein (14). Some SNPs are vital for the susceptibility and outcome of leprosy (13). Studies on IL-10 gene polymorphisms at −819 and −1082 positions within the promoter region have shown disease susceptibility and resistance between populations (15–17). Reports suggests that polymorphisms of the IL-10 promoters at positions −1082 (G/A), −819 (C/T), and −592 (C/A) are associated with the resistance against leprosy in Brazilian (18), Indian groups (15) and Columbian population (19).

Although cytokine gene polymorphism is associated with the susceptibility and resistance of a disease, but its association with cytokine production is not well studied. Therefore, the goal of current study is to investigate the association of IL-10 cytokine gene polymorphisms with Th and Treg cells mediated production of IL-10 and its association with the susceptibility and progression of leprosy in Indian population.

## Materials and Methods

### Patients and Controls

A total of 132 leprosy patients (71 males, 61 females) with bacteriological index (BI) 2–6 and 120 healthy controls were recruited from Department of Dermatovenerology, AIIMS, New Delhi, India (Table 1). The study was approved by the Institute Ethics Committee, All India Institute of Medical Sciences (AIIMS), New Delhi. Patients and age matched healthy volunteers were recruited after obtaining their informed consent. All patients were without any history of mycobacterial, HIV or any other infection and belonging to the same geographical region as the patients were included and healthy controls.

**TABLE 1:** Characteristics of the study population.

	Leprosy patients (*n* = 132)	Controls (*n* = 120)
**Age (years)**	***n* (%)**	***n* (%)**
Mean	38.4 ± 15.3	32.2 ± 13.1
**Gender**		
Male	71 (53.8)	74 (61.7)
Female	61 (46.2)	46 (38.3)

*Leprosy patients were classified according to Ridley–Jopling classification.*

### Genotyping of SNPs for Cytokine Genes

Ten milliliters blood from each leprosy patients and healthy control was collected. 5 mL of blood in heparinized vials were subjected to DNA isolation and PCR–SSP assay, while and another 5 ml collected for IL-10 ELISA and flowcytometry. DNA was extracted by using phenol–chloroform method and stored at −80°C till further use. DNA was quantified by NanoDrop1000 spectrophotometer (Thermo Scientific NanoDrop 1000; NanoDrop Technologies, Wilmington, DE, United States) and samples having 260/280 ratio in the range of 1.82–1.86 were used for the PCR–SSP analysis. Subsequently, DNA was subjected to PCR amplification using Cytokine CTS–PCR–SSP Tray Kit (Heidelberg, Germany, United Kingdom) according to manufacturer’s instructions and data was analyzed as described in earlier report (16) (Supplementary Figure S1).

### ELISA

IL-10 cytokine level was quantified by ELISA (Ready Set Go, eBioscience, San Diego, CA, United States) as per manufacturer’s instructions. Serum samples were tested in duplicates in a 96-wells plate (Nunc, Rochester, NY, United States) precoated with biotin conjugated anti-human antibodies IL-10. Protocol was followed according to the manufacturer’s instructions and optical density of each well was measured at 450 nm.

### PBMCs Isolation and Flow Cytometry

Blood samples were layered on ficoll-hypaque (Sigma Aldrich, United States), and mononuclear cells were isolated by centrifugation at 1,500 rpm for 25 min. Cells were washed thrice in sterile phosphate buffer saline (PBS) by centrifugation at 1,500 rpm for 10 min. Washed cells were resuspended in RPMI 1640 along with 10% fetal calf serum (Gibco, CA, United States) and cell viability and enumeration were estimated by 0.2% trypan blue using hemocytometer. 1 × 10^6^ cells/ml were stimulated with *M. leprae* sonicated antigen (10 μg/ml) kindly provided by Dr. P. J. Brennan of Colorado State University. All the cultures were stimulated with IL-2, anti-CD3/CD28. After stimulation, cultures were incubated in 5% CO_2_ incubator at 37°C for 48 h. After 48 h, cultured cells were harvested and stained with surface antibodies Alexa Fluor 488 Mouse Anti-Human CD4 (RPA-T4), APC-Cy7 Mouse Anti-Human CD25 (Clone: M-A251) for 60 min at 4°C in the dark. After surface staining, cells were incubated with intracellular staining buffer for 15 min at room temperature; cells were washed twice and permeabilized with 1X permeabilization buffer (eBioscience, United States) 30 min at room temperature. The cells were washed twice, resuspended in staining buffer, and incubated with PE Rat Anti-Human IL-10 (Clone: JES3-19F1). Staining was performed according to the specifications of the manufacturer. All the antibodies were obtained from BD Biosciences, San Diego, CA, United States. The cells were fixed in 400 μl of 2% paraformaldehyde and stored at 4°C. For intracellular staining, cultures were incubated with Protein Transport Inhibitor containing Monensin (BD Golgi Stop) for 4 h prior to harvest to block the secretion of cytokines. The data were collected using FACS Canto flow cytometer (BD Biosciences, United States) and analyzed cytometry along with isotype controls of phycoerythrin (PE mouse IgG1), Alexa Fluor 488 (mouse IgG1), APC Cy7 (mouse IgG1) using FlowJo software. Details of gating strategy are provided in Supplementary Figure S2.

### Immunohistochemistry (IHC)

Five micrometer thick formalin fixed paraffin embedded tissues were cut by rotary microtome (Leica Biosystems Nussloch, Germany, United Kingdom). The sections were picked-up on poly L-lysine (Sigma Aldrich, St. Louis, MO, United States) coated glass slides and stored at room temperature. Rabbit anti human IL-10 antibody (Abcam, CA, United Kingdom) was used in the dilution of 1:100. Briefly, sections were first deparaffinized, followed by rehydration and blocking of endogenous peroxidase activity by 3% H_2_O_2_ and antigen-retrieval with Tris-EDTA (pH-9.0) buffer. The sections were incubated with 1% albumin for 1 h for blocking. After three washes with PBS, sections were incubated with anti-human IL-10 overnight at 4°C in humidified chamber. The sections were again subjected to three washes of PBS followed by incubation with secondary antibody anti rabbit IgG (VECTASTAIN^®^ ABC-HRP Kit) for 30 min. Color was developed using diaminobenzidine (DAB1) chromogen system. Positive and negative stained cells were counted under the microscope using Image Pro express 6.0 software (Media cybernetics, United States) and percentage calculated from multiple fields.

### Statistical Analysis

Genotype frequencies were obtained through direct counting. Data was analyzed using statistical software Graph-Pad Prism (San Diego, CA, United States). All categorical variables were expressed in *n* (%) and continuous variables were expressed in Mean ± SD. ANOVA was applied to compare the continuous variables between multiple groups; whereas *t*-test was applied to compare the two groups. Proportions of categorical variables among groups were compared using chi-Square test. Logistic regression analysis was used to calculate the Odds Ratio (OR). A *p*-value of <0.05 was considered to be statistically significant.

## Results

### Association Between IL-10 (-819) CT/TT Genotypes With Leprosy Susceptibility

Mutations occurred more often in the IL-10 (anti-inflammatory cytokine) promoter gene than in the TNF-α (pro-inflammatory) promoter gene in all leprosy groups (18). Genotype distribution for IL-10 −819 (*C/T at −819: rs1800871)* are shown in Table 2. A significantly higher frequency of −819 TT genotype was observed in leprosy patients (59.8%) as compared to healthy controls (27.5%) (*p* < 0.0001, OR = 0.25, 95% CI 0.149–0.432). Contrasting to this, leprosy patients (7.6%) had significantly lower frequency of −819 CC genotype than the control subjects (20.8%) (*p* = 0.002, OR = 3.21, 95% CI 1.47–7.01). Genotype distribution analysis also revealed that 32.6% of leprosy patients and 51.7% of control subjects had −819 CT genotype (*p* = 0.002, OR = 2.21, 95% CI 1.31–3.68). These results demonstrate that substitution of C allele by T allele make the individuals susceptible toward the leprosy disease. As shown in Table 2, TT genotype has higher bacteriological index (BI: 4.2-6) as compared to the CC genotype (BI: 3-4). These data corelates BI with TT genotypes and pointing out that TT genotypes are susceptible and CC genotypes are resistance toward the *M. leprae* infection.

**TABLE 2:** Distribution of IL-10 (*C/T at −819: rs1800871*) genotypes in leprosy patients and control subjects.

SNPs	Genotype	Controls	Patients	BI	OR (*P*-value)	CI
IL10-^819^	CC	25 (20.8)	10 (7.6)	3.0–4.0	3.21 (0.002**)	1.470–7.011
	CT	62 (51.7)	43 (32.6)	2.5–4.5	2.21 (0.002**)	1.328–3.687
	TT	33 (27.5)	79 (59.8)	4.5–6.0	0.25 (<0.0001****)	0.149–0.432

χ^2^ test with contingency table for genotype between leprosy patients and control subjects, bacteriological index (BI), odds ratio (OR), p** > 0.005; p**** > 0.0005 and confidence interval (CI).

### Association Between IL-10 (-1082) GA/GG Genotypes and Leprosy Susceptibility and Resistance

Notably, a significantly higher frequency of the homozygous −1082 GG (*G/A at −1082: rs1800896)* genotype was observed when leprosy patients were compared with control subjects (25.8 vs. 9.2%) (*p* = 0.0006, OR = 0.29, 95% CI 0.139–0.605). Moreover, frequency of homozygous GA genotype was lower compared between leprosy patients and healthy controls at −1082GA position. We found a lower frequency in patient group (37.9%) compared to control group (40.8%) (*p* = 0.698, OR = 1.13, 95% CI 0.682–1.88). When leprosy patients (36.5%) were compared with controls (50%) at −1082AA position, significant difference was observed (*p* = 0.03, OR = 1.75, 95% CI 1.05–2.89) (Table 3). Higher bacteriological index (4.8–6) in GG genotype as compared to AA genotype (2–3.2) indicates its susceptibility toward leprosy as compared to AA genotype that showed resistance.

**TABLE 3:** Distribution of IL-10 (*G/A at −1082: rs1800896*) genotypes in leprosy patients and control subjects.

SNPs	Genotype	Controls	Patients	BI	OR (*P*-value)	CI
IL10-^1082^	GG	11 (9.2)	34 (25.8)	4.8–6.0	0.29 (0.0006***)	0.140–0.605
	GA	49 (40.8)	50 (37.9)	3.0–4.5	1.13 (0.6987)	0.682–1.878
	AA	60 (50.0)	48 (36.5)	2.0–3.2	1.75 (<0.0289*)	1.057–2.897

χ^2^ test with contingency table for genotype between leprosy patients and control subjects, bacteriological index (BI), odds ratio (OR), *p < 0.05; ***p < 0.0005 and confidence interval (CI).

### 819TT Genotypes Are Higher Producer of T Helper Cells (CD4^+^IL-10^+^) IL-10 in Leprosy Patients

**FIGURE 1:**
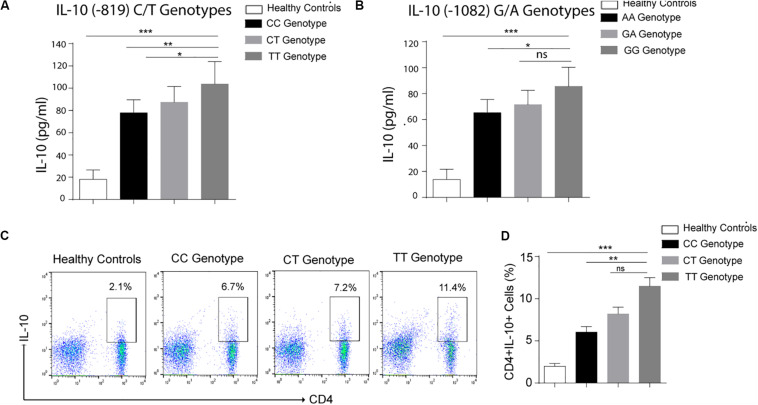
Graph plot is showing IL-10 level in the serum of leprosy patients produced by various genotypes **(A)** Healthy controls (HCs), −819 CC, CT and TT genotypes **(B)** Healthy controls (HCs), 1082 AA, GA, and GG genotypes. **(C)** Representative FACS plot showing enumeration of T helper cell producing IL-10 (CD4^+^IL-10^+^) frequency in peripheral blood mononuclear cells isolated from leprosy patients with various genotypes (-819CC, CT and TT) and healthy controls (HCs) cultured with *M. leprae* sonicated antigen for 48 h. **(D)** Graph Plots are showing total interleukin-10 (IL-10) producing CD4^+^ cells by various genotype (-819CC, CT and TT) in leprosy patients (*n* = 10) and healthy controls (HCs),Mean ± SD values are shown in each set while *p* value <0.05 was considered significant. 100,000 cells were acquired and analyzed by flow cytometry. Data analysis was performed with flowjo software. Statistical analysis was done using ANOVA test (*p < 0.05; **p < 0.005; ***p < 0.0005).

IL-10 is an anti-inflammatory cytokine produced by several immune cells like monocytes and macrophages, T cells, Treg cells as well as Breg cells (20). To find out the association of cytokine gene polymorphism with IL-10 production by T helper cells, we performed flowcytometry using CD4 and IL-10 antibodies. As shown in Figures 1C,D, IL-10 production by CD4^+^ was affected by cytokine gene polymorphism at −819 position. IL-10 levels produced by CD4 cells were significantly higher in TT genotype as compared to CC genotype. CT genotype was also higher producer of IL-10 as compared to CC genotype, but lower than the TT genotype. These results suggest that the substitution of C by T allele does affect the IL-10 production by CD4 cells in leprosy patients. Increased IL-10 levels in serum of TT bearing genotype at IL-10^–819^ position as compared to CC genotype in leprosy patients further complemented these results (Figures 1A,B).

### Increased Production of IL-10 in −819TT Genotypes by T Regulatory Cells (CD4^+^CD25^+^IL-10^+^) in Leprosy Patients

**FIGURE 2:**
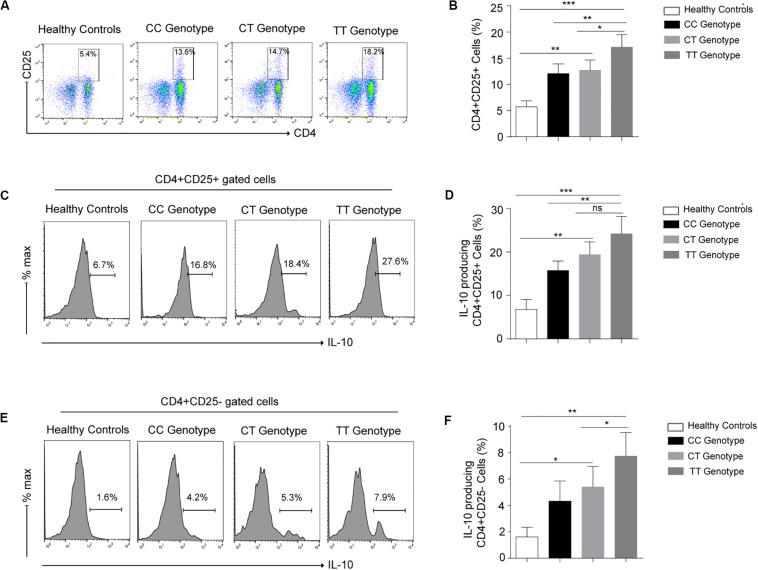
**(A)** Representative cartoon showing T regulatory cells (CD4^+^CD25^+^) cells in peripheral blood mononuclear cells (PBMCs) isolated from peripheral blood of various genotypes (-819CC, CT and TT) of leprosy patient (*n* = 10) and healthy controls (HCs). **(B)** Graph plot is showing the percentage of T regulatory cells (CD4^+^CD25^+^) in various genotypes (-819CC, CT and TT) in leprosy patients and healthy controls (HCs). **(C)** Representative histogram showing expression of IL-10 in Treg (CD4^+^CD25^+^) cells in PBMCs isolated from peripheral blood of various genotypes of leprosy patient and healthy controls (HCs). **(D)** Graph plot is showing expression of IL-10 in Tregs in leprosy patients by various genotype and healthy controls (HCs). **(E)** Representative histogram showing secreation of IL-10 by (CD4^+^CD25^–^) cells in PBMCs isolated from peripheral blood of various genotypes of leprosy patient and healthy controls (HCs). **(F)** Graph plot is showing production of IL-10 by (CD4^+^CD25^–^) cells in leprosy patients by various genotype and healthy controls (HCs). Mean ± SD values are shown in each set while *p* value <0.05 was considered significant. 100,000 cells were acquired and analyzed by flow cytometry. Data analysis was performed with flowjo software. Statistical analysis was done using ANOVA test (*p < 0.05; **p < 0.005; ***p < 0.0005).

To identify the frequency of Treg cells in various (CC, CT, TT) genotypes at −819 position in leprosy patients, immunophenotyping was done by using CD4, CD25 markers (Figure 2A) in PBMCs isolated from leprosy patients. Treg cells was profoundly augmented in TT genotype as compared to CC and CT genotypes in leprosy patients (Figures 2A,B). Increased Treg population in TT genotype patients suggested their important role in the host immune suppression observed in TT genotypes. To evaluate the functional enrichment of Treg cells in various genotypes (CC, CT and TT) in leprosy patients, we measured intracellular IL-10 production by flow cytometry (Figure 2C), using PBMCs derived from leprosy patients. Percentage of IL-10 producing Treg cells (CD4 + CD25 +) was significantly higher among TT genotypes in leprosy patients (Figures 2C,D) compared to that of CC and CT genotypes of leprosy patients (*P* = 0.0003). The augmentation of IL-10 producing Treg cells in TT genotypes directs their suppressive role by releasing IL-10. We also observed the simmiler pattern of IL-10 production by CD4^+^CD25^–^ cells in various genotypes (CC, CT and TT) of leprosy patients (Figures 2E,F).

### Higher Expression of IL-10 in the Skin of IL-10 (-819) TT Genotype of Leprosy Patients

**FIGURE 3:**
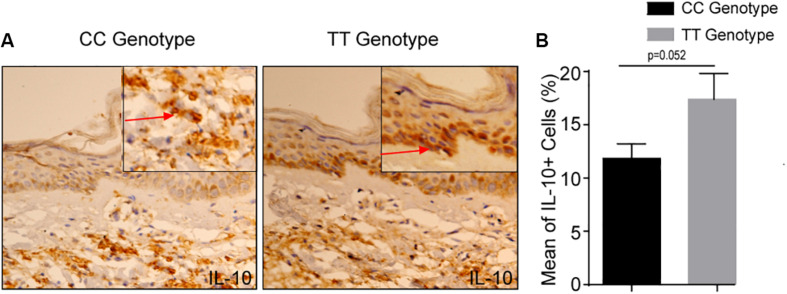
**(A)** Increased IL-10 + cells in −819TT genotype in leprosy skin lesions. Immunohistochemistry on representative skin lesions from CC and TT genotypes of leprosy patients (*n* = 5). **(B)** Graph diagram showing increase of cells with IL-10^+^ in TT genotype of leprosy patients as compared to CC genotype at −819 position. Bars indicate Mean ± SD of positive cells of 1,000 total cells. Statistical analysis was done using Student’s *t*-test for unpaired samples (*p < 0.05).

Expression of the IL-10 also measured in skin of leprosy patients bearing TT and CC genotypes at −819 position. The expression of the IL-10, was also significantly higher in the TT genotypes of leprosy patients as compared to CC genotype (Figures 3A,B). This again highlights that IL-10 producing immune cells are possibly recruited at the lesioned sites (which is more in TT genotype) of leprosy patients, where they facilitate their characteristic suppressive action. These results suggested that cytokine gene polymorphism at −819 position are associated with IL-10 production in leprosy patients and makes more susceptible toward leprosy.

## Discussion

During leprosy, there is a full-blown systemic activation of immune responses. As a result, leprosy is escorted by altered immune and imbalanced cytokine responses, which ultimately leads to excessive inflammation. Cytokines modulates the immune response of a host but cytokine gene polymorphism affects the cytokine production. This is the underlying reason to the distinct individual-specific immune responses. The idea behind studying the cytokine polymorphism in leprosy patients was: hypothetically a change either in regulatory region (promoter) or coding region is expected to modulate the expression of cytokines. A specific genotype either may result in low or high production of respective cytokine, which in turn could eventually alter the direction of immune response. Increased IL-10 production has been observed in leprosy patients and up-regulation of IL-10 is a vital mechanism in the suppression of T cell-driven immune response (21). SNPs in IL-10 in the distal and the proximal regions are formed of haplotypes in the promoter gene, and such haplotypes were found to be associated with differential secretion of IL-10 cytokine (22). Our present data demonstrates that IL-10 −819 TT genotype frequency was greater among the leprosy patients than the control subjects. Frequency of GG genotype at IL-10 −1082 position was also higher in the leprosy patients as compared to healthy control. These results corroborate with our previous findings (16) and it has also been reported that the individuals in Brazilian population bearing IL10 −819 CC genotype were resistant to leprosy (18). Our results are further confirming the results of earlier findings that −819T allele and −819TT genotype were associated with leprosy susceptibility (17) in Indian population. These findings suggested that substitution of C by T at −819 in IL-10 promotor may be involved in differential cell mediated immune response in leprosy patients. It may also influence on the production of IL-10 levels as well as makes the individual susceptible toward leprosy. Moreover, a study by Malhotra et al. (17) suggested that −819TT and −1082GG are the most frequent genotype associated with susceptibility of leprosy. In our study, homozygous IL-10 −1082 GG genotype was significantly higher in leprosy patients compared to the controls, due to the predominance of G allele suggesting an impact for allele G in leprosy susceptibility. Similar findings have been observed by other groups, where they found that the extended genotype 1082GG, 819TT was associated with leprosy susceptibility (15). Moreover, another study by Pereira et al. demonstrated that −1082G/G genotype reinforced the results, indicating that the combination carrying −819TT and −1082G/G was associated with leprosy susceptibility (23).

To evaluate the association of cytokine gene polymorphism on its function, we tested leprosy patients and quantified IL-10 levels in various genotype −819 C/T and −1082 G/A. We observed elevated levels of IL-10 in −819TT and −1082GG genotypes in the serum of leprosy patients as compared to other genotypes. Thus, −819TT and −1082GG bearing genotypes were high IL-10 producers. These findings brought up a new direction to understand the pathogenesis of leprosy. These sustained high levels of IL-10 are necessary to lead leprosy to a chronic and T cells unresponsive state. Leprosy patient presents a state where genetic mosaicism is associated with cytokine production that dictates various immune reactions, resulting to ambiguous interpretations linked with the different clinical outcomes. IL-10 produced by various immune cells such as Tregs, Th2, Bregs and other cells suppresses the immune system of host and responsible for T cell anergy in leprosy patients (24–26). We want to address the source of IL-10 production by various genotypes in leprosy patients. To answer this, we have evaluated the expression of IL-10 in T helper and Treg cells of leprosy patients. Remarkably, IL-10 −819 TT and IL-10 −1082 GG genotypes were appeared as high producer of IL-10 by T helper as well as T regulatory cells. Increased level of IL-10 was found to be logical and supported our previous reports (12, 16). This data designates that high amount of IL-10 in the microenvironment suppresses the host immune system, that eventually may help in expansion of *M. leprae* continually in leprosy patients, as high bacteriological index correlates with high level of IL-10 in −819TT and −1082 GG genotypes. Therefore, it can be inferred here that the patients with −819TT and −1082 GG genotypes are bearing higher risks of the growth of *M. leprae.*

Since leprosy is a chronic inflammatory disease and its severity is associated to the host immune response, and the level of IL-10 production can be vital to define disease outcome. Our data point-outs the conclusive association of the −819TT and −1082 GG genotypes with the susceptibility of leprosy and suggests that these polymorphisms have remarkable role in higher production of IL-10 by T helper and T regulatory cells.

## Data Availability Statement

The datasets presented in this study can be found in online repositories. The names of the repository/repositories and accession number(s) can be found in the article/ Supplementary Material.

## Ethics Statement

The studies involving human participants were reviewed and approved by The Institute Ethics Committee, All India Institute of Medical Sciences (AIIMS), New Delhi, India (IESC/T-417/01.11.2013). The patients/participants provided their written informed consent to participate in this study.

## Author Contributions

MT, HN, and CS designed experiments. MT, HS, and CS performed experiments and analyzed the data. MT, HN, and MS wrote the manuscript. NK contributed to clinical diagnosis. AS, HS, and CS critically reviewed the manuscript. All authors discussed the results and contributed to the final manuscript and reviewed manuscript.

## Conflict of Interest

The authors declare that the research was conducted in the absence of any commercial or financial relationships that could be construed as a potential conflict of interest.
