# Supplementary figures and images for: Association of IL-10 Gene Polymorphism With IL-10 Secretion by CD4 and T Regulatory Cells in Human Leprosy
Source: Front Immunol. 2020 Aug 6;11:1974. doi: 10.3389/fimmu.2020.01974 (PMC7424005; doi:10.3389/fimmu.2020.01974)

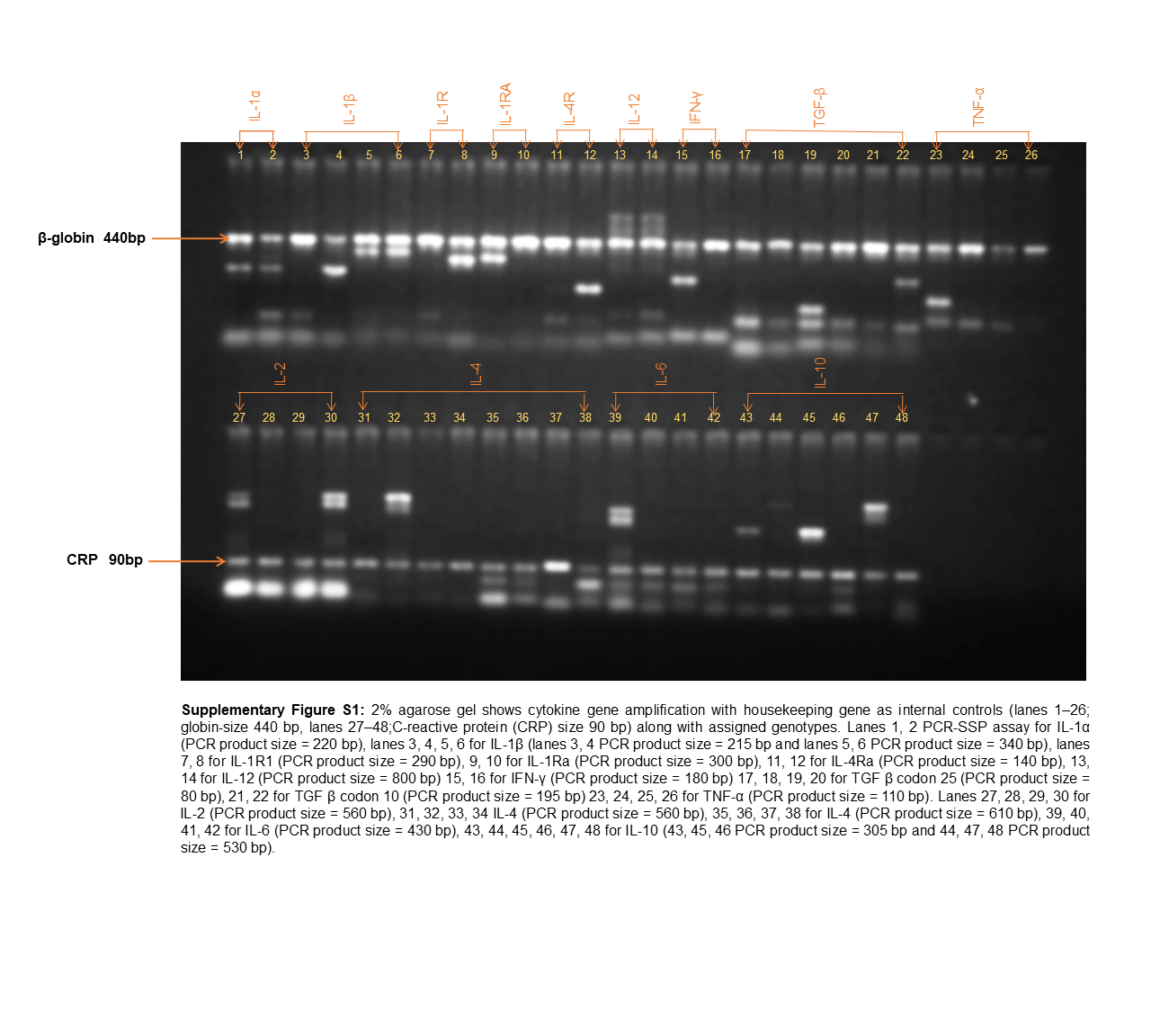

Supplement: Supplementary file 1 [file Image_1.TIF]

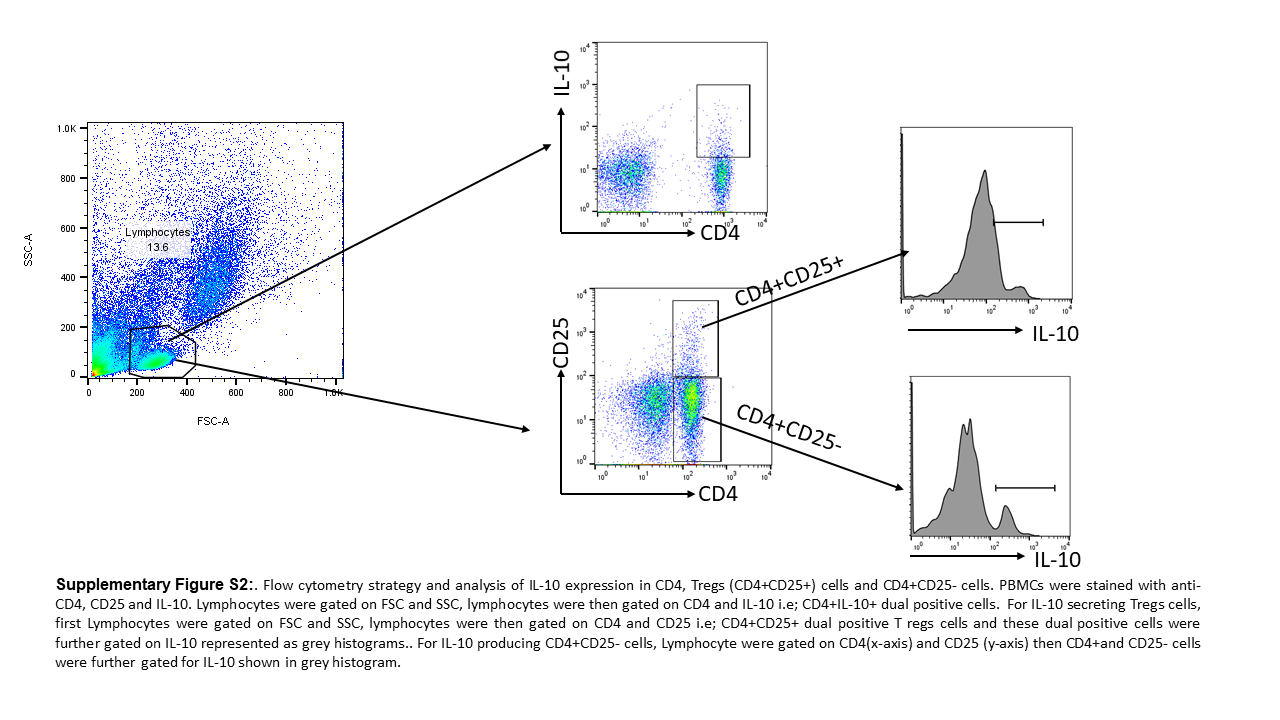

Supplement: Supplementary file 2 [file Image_2.TIF]
